# The Brussels Effect, European Regulatory Power and Political Capital: Evidence for Mutually Reinforcing Internal and External Dimensions of the Brussels Effect from the European Digital Policy Debate

**DOI:** 10.1007/s44206-022-00031-1

**Published:** 2023-01-23

**Authors:** Annegret Bendiek, Isabella Stuerzer

**Affiliations:** 1 German Institute for International and Security Affairs (SWP), EU/Europe Research Division, Berlin, Germany; 2 Ludwig Maximilian University of Munich, Munich, Germany

**Keywords:** Brussels effect, Digital sovereignty, European integration, Norms externalisation, EU-US Trade and Technology Council

## Abstract

Anu Bradford has described the European Union’s ability to externalise its norms and standards as the so-called Brussels effect. We apply the Brussels effect to select issues discussed via the EU-US Trade and Technology Council (TTC) and show that its capacity to project power stems not only from the EU’s market size but also from its domestic decision-making structure. The political capital accumulated in the EU’s consensus-based and inclusive deliberations functions as an effective instrument for motivating other states to adopt European regulations, as legislative acts resulting from European inter-institutional and multi-level policy-making hold high standards of legal certainty and signal European strategic goals and political commitments credibly. Knowing that European consensus is an important condition for externalisation, Brussels can facilitate consensus by calling for internal compromise in order to be able to take the European compromise to the international stage. Thus, the internal and external dimensions of the Brussels effect are mutually reinforcing. This twofold appearance demarcates it from the California effect and the Beijing effect.

## Introduction

In 2012, Anu Bradford observed that the European Union (EU) has a “strong and growing ability to promulgate regulations that become entrenched in the legal frameworks of developed and developing markets alike, leading to a notable ‘Europeanization’ of many important aspects of global commerce.” She calls this an “unprecedented and deeply underestimated” regulatory power that the EU is able to exercise via its “legal institutions and standards.” Bradford coined the term “Brussels effect” (2012) to describe this European ability to exercise power beyond its borders as well as its mechanism of setting standards and then requiring compliance with these standards to gain or have continued access to the European single market, a significant marketplace and economic player in global affairs. In her 2012 article, Bradford cites antitrust laws, privacy regulation, regulation of chemicals for health protection, environmental protection, and food safety as examples and focuses on legal and ideological differences between the EU and the United States (US) as well as the European ability to efficiently influence US standards. In a full monograph on the Brussels effect — subtitled “How the European Union rules the world” — that followed in 2020, Bradford additionally introduces case studies that see the EU successfully externalising its norms and principles in the digital policy arena. Drawing on Bradford’s findings, we argue that the Brussels effect also has a previously overlooked domestic dimension: the Europeanisation of international standards and third parties’ regulatory policies provides incentives to the member states to comply with and agree on European regulations. The European Commission is able to use its international regulatory power for supporting its domestic political agenda of deepening integration and can further restore European sovereignty in the digital realm despite a lack of leading technology companies or a European “Silicon Valley”. This process of European re-sovereignisation has been underway since 2013 when policy-makers from France first cautioned that Europe was at risk of becoming a “digital colony” of its powerful Western ally, the US, and was hence suffering from a loss of importance, or even sovereignty, as an international actor between US technological prowess and Chinese hegemonic aspirations (Nocetti, 2019). In response to these challenges, the European Commission has worked to reduce “virtual borders” (European Economic and Social Committee, 2017) and strengthen digital (market) integration, hence increasing internal re-sovereignisation, so that it can leverage the Brussels effect to advance external re-sovereignisation as well. In view of diverse challenges — from protecting critical infrastructure and safeguarding civil liberties to the creation of common markets — “positive integration”, meaning targeted EU regulatory action, is the way to tackle market failure within and beyond Europe (Scharpf, 1999) as regulations at the EU level take effect inside and outside the internal market given the fact that access barriers to the EU single market are often the highest global standard required for market entry. The EU is able to ensure such standards by leveraging its regulatory power, that is, making use of the Brussels effect. Given that digital integration is a precondition for asserting European standards and norms externally, and therefore constitutes an integral element of European Digital Foreign Policy, the Brussels effect arguably has a previously overlooked internal dimension of incentivising European integration in addition to its often-discussed external dimension of internationalising European standards.

For making our case of mutually reinforcing internal and external dimensions of the Brussels effect, we produce evidence from the European Union’s digital foreign policy strategy, more specifically, we are looking at select policy issues discussed in the context of the EU-US Trade and Technology Council (TTC). We find that the Brussels effect is not only rooted in market power but also its endogenous effect of deepening European integration. This endogenous effect comes to pass as follows: European policy-makers are aware of the Brussels effect, and in order to be able to externalise Europeans norms, an internal agreement on these norms has to be reached first. International partners trust that legislative acts resulting from European deliberations provide high legal certainty once adopted. Thus, Brussels can facilitate consensus by calling for internal compromise in order to be able to take such a European compromise to the international stage, and hence the internal and external dimensions of the Brussels effect are mutually reinforcing.

We further hold that the Brussels effect can be distinguished from the California effect insofar that it promotes European re-sovereignisation internally and externally whereas the California effect simply reflects the power of California as a state within the federal union of the US. In other words, the California effect lacks the internal dimension we claim to observe for the Brussels effect. Lastly, we also address the “Beijing effect” and argue that there are two main differences between the Brussels effect and the Beijing effect. Firstly, the Brussels effect impacts both private and global players as well as major powers whereas the Beijing effect is limited to countries that are dependent on the People’s Republic of China (PRC) economically and lack technological know-how and human capital. Secondly, the Brussels effect is rooted in democratic values and the consensus-driven decision-making procedure of the EU whereas China is ruled by the Communist Party of China (CPC) which has digital geostrategic ambitions but no intention of promoting the idea of informational self-determination.

## Understanding Nature and Dimensions of EU Digital Sovereignty

The Brussels effect describes a mechanism that sees the European Union externalising its norms via its “legal institutions and standards” (Bradford, 2012). For illustrating this mechanism in regard to EU digital sovereignty, we will lay out the digital strategy of the EU, detail its core concepts and strategic goals and discuss what internal and external challenges this strategy encounters through the lens of the political and analytical merit of the Brussels effect.

The European Commission has declared the years 2020–2030 Europe’s “digital decade” and identified securing European “technological sovereignty” and “digital sovereignty” as key strategic goals for this period. These terms were first used by industry representatives who cautioned that industrialised European nations were dependent on the availability, integrity and confidentiality of current and emerging technologies, both for civilian and safety purposes; furthermore, they cautioned that Europe was lacking production capacities and R&D investments (Bonß, 2021). Given that many concerns regarding the vulnerability of critical technological infrastructure are often also discussed as cybersecurity issues, the term digital sovereignty is sometimes used interchangeably with “technological sovereignty”. In a 2020 strategy paper, the European Parliament defines digital sovereignty as “Europe’s ability to act independently in the digital world” and highlighted this issue’s importance as “[s]trong concerns have been raised over the economic and social influence of non-EU technology companies” (European Parliamentary Research Service, 2020). EU citizens’ control over their personal data is endangered and “the growth of EU high-technology companies and the ability of national and EU rule-makers to enforce their laws” are constrained (European Parliamentary Research Service, 2020) when EU digital sovereignty is in jeopardy.

Adopted legislative acts are often considered to be the core of a modern understanding of European sovereignty. For the EU, the concept of sovereignty is better understood as a process, not a status quo. It refers to EU actors’ moderating capacity of legitimising their positions through transparent, internal opinion-forming processes along the legislative train schedule and exercising them internationally in multi-stakeholder bodies and institutions (Bendiek, 2021). The European debate on norms harmonisation and subsequent standardisation contributes to deepening integration and thus advances internal re-sovereignisation. Hence, European rules and laws which have been agreed upon among 27 member states also carry significant political capital as they are hard to unbundle in international negotiations and thus project the power of sunk negotiation costs onto the EU’s partners (Dluhosch & Ziegler, 2011). This political capital can therefore be transformed into the successful externalisation of European norms and standards.

The European Commission has strengthened not only its internal but also its external position by initiating laws addressing the regulation of new and emerging digital technologies. For instance, the European Commission takes great pride in the fact that the European General Data Protection Regulation (GDPR) and its provisions shaped not only the terms of service of leading social media platforms, including the platforms operated by Meta, but even impacted the data protection legislative debate in the US. At the same time, it is important to note that while the EU “has become a sort of ‘regulatory superpower’” (Torreblanca & Jorge Ricart, 2022), it does not see its regulatory power as a route to dominating or even eclipsing leading technology powers, especially its ally the US. However, the pursuit of digital sovereignty is challenged by external and internal factors: externally, the Covid-19 pandemic has revealed the dependency on Asian manufacturers often inhibited by harsh anti-Covid restrictions such as production stops and lockdowns. The Russian attack on Ukraine has further highlighted the vulnerability of value chains, especially the food value chain, and additionally underscored that Europe lacks capacities in the hard and military security arena. Internally, member states across Europe are concerned that the French Republic and the Federal Republic of Germany might find protectionist policies a sensible way to boost European industrial capacities (which would majorly benefit German and French companies and industrial regions) while the European Commission prefers the concept of “open strategic autonomy” (Torreblanca & Jorge Ricart, 2022).

Indeed, when presenting her college of commissioners on November 27, 2019, Ursula von der Leyen defined being a “geopolitical Commission” dedicated to multilateralism as a priority of the 2019–2024 European Commission. Since late 2019, the world has changed profoundly: the Covid-19 pandemic, intensifying Great Power competition, and the Russian attack on Ukraine have heightened threat awareness and shifted priorities in the European foreign and security strategy. The EU’s strategic narrative “strategic autonomy” describing the aim to address security challenges in hard security, value chain integrity or environmental security independently has since evolved into “open strategic autonomy”. This broadening of the concept of “strategic autonomy” resulted from the understanding that transatlantic cooperation is not only important for guaranteeing European security, but necessary, as demonstrated by the dangerous value chain bottlenecks revealeds during the Covid-19 pandemic and the Russian attack on Ukraine (Erlanger, 2020). EU (digital) sovereignty can only be realised in cooperation of like-minded countries (such as the US).

## Beyond Market Power: the Brussels Effect and European Internal Re-sovereignisation

The European Commission aims to further progress European re-sovereignisation and has understood that (digital) sovereignty can only be realised in cooperation with the US. It had already proposed an EU-US Trade and Technology Council (TTC) in mid-2020 to find common ground on trade and technology standards after a contentious relationship and disagreements with the US on economic policies during most of the Trump Administration. While this suggestion received only little attention then, the Biden Administration showed greater interest in cooperating with the EU and exploring the idea of an alliance on “democratic technology”. The TTC held its inaugural meeting in Pittsburgh, Pennsylvania, on 29 September 2021. Ten working groups of the TTC have been established, and a second meeting has been held in France in May 2022, which has produced an ambitious agenda and detailed strategy (O’Brien, 2022). In the following paragraphs, the policy arenas cybersecurity, semiconductor value chain resilience, digital market regulation and data protection regulation will be examined in order to understand how the EU seeks “to reduce vulnerabilities and interference” (Torreblanca & Jorge Ricart, 2022) by externalising its regulations via the TTC. We show how this strategy contributes to strengthening EU internal and external digital and technological sovereignty and use it to illustrate the mutually reinforcing nature of the Brussels effect’s internal and external dimensions.

### Cybersecurity and Connectivity

An important topic on the agenda of the TTC has been debate whether to include or exclude the Chinese company Huawei from national telecommunications infrastructure development. Huawei was the world’s first company to be able to build and run infrastructure based on the 5G standard and has expressed interest to invest in connectivity development in Europe — increasing connectivity at high quality and affordable costs is an important goal across European regions. However, Huawei is also controlled by the Chinese government through an opaque ownership structure, which causes concerns for allowing the company to build and operate infrastructure that can be exploited for gaining access to information ranging from confidential IP such as trade secrets to information sensitive for national security (Niquet, 2018). By introducing key European cybersecurity legislation and taking a closer look at the case of Huawei investments in the EU, we show the strength of the internal dimension of the Brussels effect lies in its qualities of signalling European unity and credibility, as the relevant laws result from a long and consensus-based legislative process, which makes them credible and durable. Furthermore, they express a long-term European commitment even if select member states take longer to translate specific regulations in national law.

Concerns regarding the cybersecurity of information networks and telecommunications equipment can be discussed in the EU via the forum the Cooperation Group of the Network and Information Security Directive (NIS Directive) offers. The NIS Directive was introduced in 2016 and is the key building block of EU cybersecurity policies. It consists of three parts — national capabilities, cross-border collaboration and national supervision of critical sectors. The NIS entails a first set of international standards in the cyber realm for accession to the European market (Directive (EU) 2016/1148). Since 2021, the proposal for a NIS 2.0 Directive and the mandate of the Committee on Industry, Research and Energy to enter into interinstitutional negotiations have further advanced the debate surrounding framework guidelines for European cybersecurity and demonstrated potential for harmonised EU-wide cyber regulation (COM/2020/823; Negreiro Achiaga, 2022), and a provisional agreement on NIS2 has already been reached by the co-legislators (Groothuis, 2022).

To further European cybersecurity resilience, the European Parliament adopted the EU Cybersecurity Act in 2019, which established a cybersecurity certification framework for information and telecommunication products and services that companies want to offer on the European market; certification schemes were drafted by the EU Agency for Cybersecurity (ENISA). Of special interest are the assurance levels — basic, substantial and high — that indicate the cybersecurity risk associated with purchasing and using a specific product. The 2019 Cybersecurity Act does not make the certification process mandatory yet; discretion on whether to adopt it or not lies with the European Union member states’ national governments; oversight of equipment certification is also a responsibility of the member states. The European Commission nonetheless emphasises that the certification framework has the advantage of allowing comparability across member states and thus improves reliability and trustworthiness based on one unified European benchmark (Regulation (EU) 2019/881).

Additionally, the European Commission presented a “toolbox” on secure 5G networks in January 2020. The toolbox includes strict access controls before allowing a telecommunications company to contribute to the establishment and operation of national 5G networks. The recommendation highlights that “[e]nsuring European sovereignty should be a major objective, in full respect of Europe’s values of openness and tolerance” and that “cybersecurity of 5G networks is key for ensuring the strategic autonomy of the Union”. As the name already suggests, this toolbox is also only intended as a set of recommended best practices and potential tools to improve 5G network security in individual EU member states and is not legally binding (NIS Cooperation Group, 2020). Still, the recommendations included in the toolbox are very precise, as they include strict access controls before allowing a telecommunications company to contribute to the establishment and operation of national 5G networks. They even directly encourage “necessary exclusions” in the case a supplier can be considered high-risk according to the EU-wide Coordinated Risk Assessment. The coordinated risk assessment of the cybersecurity of 5G networks was published in October 2019 and emphasises the importance of conducting a risk assessment and implementing access controls for maintaining European digital sovereignty and strategic autonomy (NIS Cooperation Group, 2019).

Lastly, the European Commission adopted a European Cyber Resilience Act with the purpose to “establish common European cyber security standards for products (especially connected objects) and services that are placed on our market,” according to Commissioner Thierry Breton (Breton, 2021). This act was first announced in 2021 and subsequently opened for input via an EU multi-stakeholder consultation process (Chee, 2022); thus, this European cybersecurity legislation is indicative of a European attempt to harmonise and further integrate the European digital market as well as to regulate access to this market.

European member states considered advancing their connectivity (especially their 5G infrastructure) with the help of Chinese manufacturer Huawei, which has ties to the CPC and the People’s Liberation Army (Niquet, 2018). For some time, the US threatened to cease sharing intelligence with any partners that relied on Huawei’s technological infrastructure for fear of information theft or infrastructure corruption (Salama, 2020). This sparked debate in Europe, as Huawei is the world’s leading developer and provider of 5G products and services, having finalized more 5G contracts than any other telecom company by 2021, 50% of which were for 5G networks in Europe (Sacks, 2021). American adamancy to exclude Huawei (and other Chinese competitors) from progressing the Open RAN alliance is another contentious issue between the transatlantic partners (Emmott, 2020). “Open RAN” (Radio Access Network) presents a “technology concept in the area of 5G mobile communications that introduces additional and open interfaces for previously proprietary components of the radio access network […] intended to promote openness and interoperability in the RAN of a mobile network” (Köpsell et al., 2022) and shall help increase independence from key ICT providers as an “alternative way” of building networks reliant on technologies such as cloud computing (Lee-Makiyama & Baker, 2022). The case of the stagnant Open RAN alliance also demonstrates that digital sovereignty cannot mean “autarky” for either the EU or the US, as the EU lacks the industrial capacities to boost innovative Open RAN solutions, while the US largely misses market-ready 5G equipment providers, such as Nokia or Ericsson. Hence, cooperation is required if both want to prevent China from dominating the alliance (Lee-Makiyama & Baker, 2022).

Huawei lobbyists have continuously sought to fight bans or dilute provisions suggested for national implementation by the EU by pitching significant investments in national infrastructure and local research hubs, including in the Netherlands, France, Italy and Spain (Cerulus, 2020). European governments and companies expressed concerns about the advancement of their digital connectivity if global market leaders such as China’s Huawei are excluded from the internal market (Lilkov, 2021) while the US Federal Communications Commission has also identified and listed five companies (all from China) whose equipment and services are deemed an unacceptable national security risk (Federal Communications Commission, 2021) and remarked that the European toolbox was not strict enough (Komaitis & Sherman, 2021). Lobbyists like to point out that some Huawei products have been awarded a Common Criteria certification, such as by the Spanish National Cryptologic Centre which awarded Huawei the world’s first EAL4 + security certificate. However, Spanish authorities have meanwhile responded to the extensive reference to this certification by Huawei company officials to support their stance that Huawei equipment did not pose a security risk by clarifying that the certification only pertains to the technical abilities of the certified Huawei equipment, and not any kind of cybersecurity requirements (Tekdeeps, 2020).

It is noteworthy that despite its competitive edge in 5G technology, China’s rise in the field of technological progress is relative, and other companies are catching up fast. By 2020, Huawei already ranked only fourth in the list of companies filing for most 5G patents, following Samsung Electronics, Nokia and LG Electronics. Furthermore, Ericsson has eclipsed Huawei as the top 5G provider according to the 2020 Technology and Innovation Country Readiness Index published by the UN Conference on Trade and Development UNCTAD. In other key digital technologies than 5G, including Artificial Intelligence, big data and blockchain technology, Chinese companies are not represented among the top providers (except for Alibaba’s blockchain technology). However, the only European company included other than Ericsson is SAP, which means that the US continues to be the leading innovator in the digital technology industry (UN Conference on Trade and Development, 2021). In fact, it was not until 2019 that Huawei was able to build a smartphone without manufacturing chips provided by the American company Qualcomm (Fitch & Strumpf, 2019). This means while Huawei equipment might not be replaceable immediately once a country decides to limit its involvement in 5G network development, feasible European and especially American alternatives exist which also possess the necessary technological know-how. The example of Huawei also shows a company that profited both from high public R&D investments and a targeted press campaign painting it as a standard-setting company and almost inevitable partner. In contrast, R&D investments in the EU have been comparatively low and several investment initiatives that were discussed in the Lisbon process yielded not the desired result, what the European Commission only seeks to remedy now during its “digital decade” (European Commission, 2021a, b).

This policy issue of 5G connectivity and cybersecurity offers interesting insights on the Brussels effect. The agreement on security standards and certification regimes in the EU can help to level the European playing field and demonstrate a show of unity towards allies, adversaries — and potential investors, even if some member states select to implement the recommendations only partially. Intel, America’s leading technology company in the field of 5G (UNCTAD, 2021), has recently announced a significant investment in the European Union. This new investment can be considered a success of a European external digital strategy based on the Brussels effect insofar that internal norms harmonisation increased European credibility — the EU is willing to fine Huawei (Bermingham, 2022) and reject investment opportunities — and in doing so generated political capital that can encourage investments from the US instead. This case also shows that European technological and digital sovereignty have a significant external dimension that does not equate to dominating other powers or seeking autarky but forging healthy relations with democratic technology providers which can help advance European connectivity and partially remedy the lack of European capacities. Additionally, the case of Huawei equipment also illustrates that companies not willing to comply with EU standards face market exclusion, such as Huawei in Sweden (Gkritsi, 2020).

### Elusive Semiconductor Value Chain Resilience

Aware of the challenge of rising demand for semiconductors triggered by digitalisation and greening of the economy (European Commission, 2022), the proposed EU Chips Act is designed to integrate national efforts into a coherent European semiconductor research strategy as the EU is facing dependencies along the semiconductor value chain: It is a net importer of necessary electrical and rare earth elements, but a net exporter of machines needed for semiconductor production (Ciani & Nardo, 2022). The EU Chips Act aims to facilitate collective action for (re-)building production capacities to reverse the trend of outsourcing semiconductor production, and thus signals a clear intention to regain industrial capacity (and hence technological sovereignty). Chips (also known as semiconductors) are critical components of digital technologies manufacturing, both in the civilian and military realms (Baraniuk, 2021). While American companies such as market leader Qualcomm design the chips, they are manufactured mostly in Taiwan — one single Taiwanese company produces 92% of the global chip supply of the most advanced chip type, creating a highly vulnerable supply chain bottleneck (Schoolov, 2022). The EU Chips Act calls for greater public investments in semiconductor R&D in Europe (Lomas, 2021), whereas the CHIPS for America Act, passed in June 2020, calls for investments in chip design R&D in the US (H.R.7178, 2020). Concerns about an emerging and counterproductive “subsidy race” have thus been voiced on both sides of the Atlantic. At the same time, a strictly US or EU focus on reclaiming technological sovereignty is unrealistic (Lewiset al., 2021). In these areas, international cooperation is inevitable, as “value chains of high-tech goods such as semiconductors are best understood as a complex network of specialised producers” (Poitiers, 2021) enabled by internationally scattered expertise and division of labour.

As discussed above, a key example highlighting European dependency on external partners is the recent high-profile announcement of US company Intel: It plans to invest up to €80 billion in the European Union over the next decade along the entire semiconductor value chain, with plans for a semiconductor production site in Germany, an R&D centre in France and manufacturing plants in Ireland, Italy, Poland and Spain (Intel, 2022). Interestingly, European companies have a monopoly on the manufacturing of equipment necessary to produce chips in the first place, which goes to show that “value chains of high-tech goods such as semiconductors are best understood as a complex network of specialised producers” as described by Niclas Poitiers (2021). Several European companies and member states have cautioned against a “politicisation” of the challenge of semiconductor acquisition and the European industrial strategy spearheaded by France’s Thierry Breton. Their concerns are driven by the fact that dependencies on chips from the US and Asia are unevenly distributed across member states, and that some member states are more dependent on Chinese FDI — a source of funding they fear to compromise should a harsh exclusion of Chinese suppliers be facilitated by Breton. Similar concerns apply to a potential negative impact on existing and planned cooperation with the US should a counterproductive subsidy race emerge which inhibits foreign R&D funding (Cerulus & Barigazzi, 2021; Le Corre, 2018; Noyan, 2021). Considering these contentious issues, the date of adoption for the EU Chips Act remains uncertain and could well be pushed back to mid-2023 (European Association of Automotive Suppliers, 2022).

Production capacities and relevant know-how for manufacturing chips themselves remain limited in Europe, as do skilled professionals or relevant education and training programs. Albeit limited compared to their competitors, there are EU semiconductor companies, which mainly focus on chips for specific sectors such as automotive and healthcare or specific niches in the supply chain, including STMicroelectronics, Infineon and NXP as well as ASML. The biggest of these companies is German semiconductor producer Infineon, which was consistently ranked 10th or 11th of the world’s biggest semiconductor companies throughout the last five years — while the three companies Samsung (South Korea), Intel (US) and TSMC (Taiwan) account for roughly half of global sales (Alsop, 2022). In terms of market share, TSMC (Taiwan) accumulates more than 50%, followed by Samsung (South Korea) with 17% and UMC (Taiwan) at 7% which in turn is followed by GlobalFoundries (US), also accounting for 7%. These top ten dominated by contract manufacturers are completed by two more Taiwanese and three Chinese companies as well as Israel’s Tower Semiconductor at 1% (Bhutada, 2021). TSMC builds chips for Intel and Qualcomm as a contract manufacturer, among others, and *Time* has estimated that it could even account for more than 90% of the advanced processors market (Campbell, 2021). Furthermore, despite Washington’s effort to counter the ever-increasing influence of the Chinese chips industry in the US and in Europe, 2021 marked the second year in a row in which Chinese chip manufacturers were the world’s biggest buyers of chip-manufacturing equipment, suggesting a flourishing and growing industry (Leonard et al., 2022). Both the US and the EU are dependent on international producers, for instance, the US and the EU account for 21% of the world’s semiconductor manufacturing capacity, but for 43% of the global consumption of digital devices, revealing a potentially dangerous dependency on Chinese manufacturers (Lewis et al., 2021).

Considering these facts, one key takeaway for understanding the merits of the Brussels effect in the case of strengthening semiconductor value chain resilience is that its internal dimension once again helps foster a European effort, for instance in distributing R&D for producing chip manufacturing equipment across Europe, even when the EU lacks leverage to shape transatlantic semiconductor value chain resilience in the fashion it desires, that is, utilising the Brussels effect’s external dimension. Time-consuming and compromise-driven debate signals European interest in and terms for cooperation with companies and international partners. The effort to develop a European semiconductor strategy and shared commitment to rebuild European capacities, even if details still remain the subject of debate, demonstrates the will to cooperate and invest internally, which constitutes political capital insofar that it signals problem awareness and readiness to act, which can help attract international investors — making the internal and external dimensions of the Brussels effect mutually reinforcing.

The fact that a set of contentious issues and resulting delay of adoption of the EU Chips Act remains also demonstrates that the European decision-making process based on consensus is very much intact despite internal divisions. This again shows that the concept of sovereignty has become even more complex and is nowadays better understood as a process, not a status quo (Bendiek, 2021). In other words, sovereignty no longer merely refers to a legally defined status — instead, it needs to be understood as a capacity for the EU to act in the European interest. The EU enacts its sovereignty by moderating internal and external partners through facilitating transnational structures of negotiations. The methods are transparent, internal opinion-forming processes reliant on consultations, e.g. with involved industries, and exercising them effectively internationally in multi-stakeholder bodies and institutions. European sovereignty cannot be equated with either national autonomy or autarky — it is the capacity to navigate and govern complex value chain processes.

### European Agenda-Setting in Digital Markets and Digital Services Regulation

The digital economy has strongly diversified over the last decade, and the personal data of private citizens themselves have become an economic good. Thus, the EU updated its rules to ensure the data sovereignty of its citizens and companies was protected and the European e-commerce directive was adopted in 2000 (directive 2000/31/EC). Now, the Digital Services Act (DSA) and Digital Markets Act (DMA) were introduced, which address issues that have arisen with the emergence of new products and service providers on the digital market (COM/2020/825; COM/2020/842). Still, the e-commerce directive remains at the heart of European digital strategy and digital foreign policy tools for regulating market access and institutionalising European norms. It sets standards for transparency requirements for service providers and liability along the business chain, including intermediary service providers and general rules for commercial communications.

The DSA introduced new rules in the issue areas of transparency, with specific information obligations on the storage and commercialisation of user data, handling hate speech and participation bans and reporting users who are found to share illegal content. The DMA is designed to establish a level playing field for enterprises in the digital age and to enable innovation and growth. It is tailored to regulate “gatekeepers”, which are defined as “large, systemic online platforms”. Examples of gatekeepers (although no companies have been designated as a gatekeeper so far) would be Amazon, Meta and Alphabet. Small and medium-sized enterprises (SMEs) depending on these gatekeepers shall be protected by the DMA, as gatekeepers can no longer utilise their power as platform providers to advertise their goods and services more prominently. Furthermore, gatekeepers are now required to allow commercial users access to data they generate while using their platforms and to allow third parties to inter-operate with their services. The data sovereignty of European shall also protected by the recently adopted Data Act, which clarifies under which conditions private data can be commercialised (COM/2022/68).

In the context of the TTC, conflicts between the partners on both sides of the Atlantic have arisen regarding the planned designation of gatekeepers, which will primarily apply to non-European companies such as social media platforms and digital marketplaces such as America’s Amazon or eBay and China’s Alibaba (Mariniello & Martins, 2021). Compliance of such companies with the provisions laid out in the DMA would mean fundamental changes to their established business models, which are based on offering free use of their platforms to private users and third commercial actors in exchange for their data and an opportunity to increase the platform’s growth. As access to the marketplaces is free, consumers can easily find an SME advertising its products there and then purchase from the SME directly rather than through the marketplace, often at a cheaper price. Thus, gatekeepers argue that they need to advertise their own products more prominently to profit as well (Meyers, 2021). While some observers caution that the definition of gatekeeper should not be too broadly interpreted and that the designation of gatekeepers should focus on companies with little competition, such as Google — which has a market share of almost 90% in Europe — American partners are concerned that US companies are specifically being targeted, and thus are calling for a broader interpretation of the term. Furthermore, US policy-makers have expressed security concerns about requiring the possibility to distribute programmes such as apps outside of “closed systems” — in other words, to install apps on smartphones and other devices without relying on the two dominating market powers, Apple (iOS) and Alphabet (Android), because this means that the cybersecurity of smart devices could be compromised by downloading malicious software from a third source without established vetting and verification processes (Stolton, 2022). This has created a dilemma for the DMA in terms of preventing discriminatory practices by market leaders while adopting non-discriminatory regulations to address data sovereignty and fair competition in the digital market.

The European Parliament approved the DSA and the DMA on 5 July 2022; the Council of the European Union followed in September 2022 (European Parliament News, 2022). Given the anticipated wide-reaching consequences of the acts entering into effect, they have been the topic of heated internal debate and intensive lobbying efforts by companies, especially from the US. Internally, the debate surrounding the DMA and DSA came to be known as a major theatre of the greater ideological conflict between Commissioners Thierry Breton and Margrethe Vestager (Larger et al., 2020). The Microsoft Cooperation has been especially active in the DSA/DMA consultation process and cautions that “the desire for speed should be carefully balanced against the need for effectiveness” (Alaily & Klynge, 2021). They also emphasise that “gatekeepers will inevitably need further guidance on how to comply with the DMA obligations” (Alaily & Klynge, 2021). Microsoft hence signalled acceptance of the rules while subtly indicating that additional guidance is needed and that such guidance should ensure that the business model of big tech corporations is not significantly affected by the DMA.

Applying the analytical lens of the Brussels effect to the genesis, provisions and contentious issues of the DMA and DSA, its potential and shortcomings as a political tool as well as the mutually reinforcing nature of its internal and external dimensions become evident yet again. Internally, there is an overall consensus that tech monopolies “must be loosened” — but to which degree and by what means is contested (Echikson, 2022). The fact that such disagreements are known is testimony to the transparency and hence legitimacy of the consensus-based European legislative debate and decision-making process across involving various European institutions. The proposals for the DMA and DSA allowed the EU to initiate negotiations with international big tech companies and to be the agenda-setter in the ensuing negotiations and consultations, such as in the TTC. The EU acted, and companies are now reacting with lobbying efforts. While this external dimension gives the EU some leverage, it certainly does not fill the big technological gap that it is confronted with, namely the lack of any competitive big tech company. Still, European regulatory power increases the political capital with which the EU enters into international negotiations in fora such as the TTC and multi-stakeholder consultation processes with actors including big tech companies. In other words, digital sovereignty as a political and foreign policy practice cannot help meet the objective of filling the technological gaps, but can aid a successful “open strategic autonomy” approach by starting international negotiations as the result of internally coordinated efforts of DSA/DMA legislation (Torreblanca & Jorge Ricart, 2022). 

### European Credibility in the Data Protection Regulation Debate

In case of the General Data Protection Regulation (GDPR), the Brussels effect even extends beyond the regulation of private actors and shapes foreign legislative debates, from Africa over Asia to the US (Cervi, 2022). The US legislative debate on a federal privacy law gained momentum after a joint call for a federal privacy law similar to the GDPR by key US players such as Apple, Alphabet, Meta and Microsoft (Pfeifle, 2018; Tiku, 2018). The involvement of dominant tech companies in this process highlights the power of the Brussels effect, as even strong market dominators such as Meta need to reconsider their terms of service and data commercialisation business model if they want to retain access to the European internal market. This is evidenced by the U-turn performed by its executive board in 2020 when key executives initially threatened to pull platforms such as Facebook and Instagram from the European market in response to the Schrems II ruling (Shead, 2022) but quickly backtracked, as this tactic did not influence the European position as desired. 25% of Meta’s revenue is generated in Europe (Kwan, 2022), which is too big of a share to lose. Consequentially, Meta had to adapt its terms to European standards and has called for a US federal privacy law that converges with the GDPR to further increase legal certainty and interoperability. Such lobbying efforts by US companies underscore the desire of private US actors to cooperate with the EU on digital and technological standards via the TTC in order to retain market access and sustain their growth. Their European counterparts are also highly involved with the TTC through formats such as the European Commission’s online consultation platform for stakeholder involvement in shaping transatlantic cooperation (European Commission, 2021a, b).

Given that digital services are “indivisible,” as Bradford puts it (2020), US companies updated their terms of service following the GDPR, which constitutes the world’s strictest and most detailed data protection regulation, as it would simply be too costly to offer a different service model across different countries. In the data protection and data flow management arena, legal certainty is currently lacking, which confirms the assumption of a mutually reinforcing nature of the Brussels effect’s internal and external dimensions, *ex negativo*, as the following paragraphs illustrate.

Data protection regulations are a key contentious issue between the EU and the US, especially since the Court of Justice of the European Union (CJEU) voided the “privacy shield” (the transatlantic agreement regulating the exchange of users’ private data between European company subsidies and their American holding companies for commercialisation purposes) in *Data Protection Commissioner v Facebook Ireland Limited and Maximilian Schrems* in July 2020 (Judgment of the Court Case C-311/18). While invalidating the Privacy Shield, the Court has ruled that international data flows can continue under GDPR provisions if they are based on EU Standard Contractual Clauses for international data transfers. The CJEU held that data exporters must verify on a case-by-case basis that the personal data being transferred will be adequately protected in the destination third country in line with the requirements of EU law. Furthermore, the CJEU stated that data exporters may implement supplementary measures to ensure the protection of personal data in destination third countries as required by EU law — however, the court did not specify what would constitute such supplemental measures. Currently, the EU and US are cooperating to find a framework replacing Privacy Shield; most recently, President Joe Biden issued an “Executive Order On Enhancing Safeguards For United States Signals Intelligence Activities” (The White House, 2022) in order to address European Concerns.

The lack of a new framework and ensuing uncertainty for European companies and consumers as well as international technology companies and service providers about what standards they have to meet to be able to conduct business in line with EU law is damaging for partners on both sides of the Atlantic. For instance, the Austrian data protection authority banned the use of the data analysis tool Google Analytics, which was a significant setback for Google but also for Austrian companies using the tool (Terharen, 2022). Following the ruling, the EU Cloud Code of Conduct General Assembly, which includes international companies, started to work on the Third Country Transfer Initiative, which seeks to address concerns regarding the processing of European users’ personal data in a third country by developing a specific “module” to complement the GDPR (European Cloud Code of Conduct, 2022). A feasible solution for providing legal certainty for transatlantic data transfers is urgently needed, as interoperability is crucial for the provision of digital services and the pursuit of further business opportunities in Europe, both by American companies and European companies working with American digital products. So far, steps discussed towards the institutionalisation of the Third Country Transfer Module have included the nomination of an oversight board, as such watchdogs and their ability to issue fines have successfully mediated company practices and GDPR regulations in the past, for instance in the cases of GDPR violations by TikTok and Meta (European Data Protection Board, 2021, 2022). However, so far no third-country module has been introduced, as it remains unclear how such a module should be designed to comply with the court’s expectations. The March 2022 agreement “in principle” on a Privacy Shield 2.0 between the EU and the US also hardly extends beyond a statement of intent to remedy the current legal state of uncertainty (Greaves & Nauwelaerts, 2022). Even though the US hoped it had a “trump card to resolve [this] long-running dispute” (Scott & Manancourt, 2022) when the Russian attack on Ukraine highlighted the urgency to increase transatlantic cooperation and reminded the EU of its dependency on the US and American intelligence-sharing in the hard security domain, the Privacy Shield 2.0 agreement has been criticised for being “useless”. Lessons from the GDPR debate and continued failure to suggest a framework for replacing the privacy shield confirms the analytical and political merit of the Brussels effect, as well as the mutually reinforcing nature of its internal and external dimensions, *ex negativo*.

While the EU has set the agenda in the GDPR debate as it has in the DMA/DSA debate, and triggered familiar responses by concerned companies in the form of declarations of intent to cooperate and lobbying efforts, it can thus far not enter into negotiations with a credible mandate, as the internal European debate has not yet produced a consensus. Results of negotiations that take place nonetheless — such as the Privacy Shield 2.0 — lack credibility and are quickly dismissed as useless (Dachwitz, 2022). The failure to produce a legally certain replacement framework hurts European companies and citizens dependent on digital services provided by American companies and damages European credibility in negotiations. Institutionalising a replacement for the privacy shield first requires a joint European effort to agree on a feasible alternative, which is only achievable through inter-level and inter-institutional debate. The replacement of the privacy shield would thus not only mean a further step in the process of European internal re-sovereignisation; it would also be an important signal reaffirming an understanding of European external digital sovereignty as sovereignty embedded in an international network of democratic partners. Clearly, for the EU, the opportunity of serving as a transatlantic agenda setter can be an advantage, but one that comes with the burden of suggesting viable tactics and tools to flesh out an agreement that works for both sides: the US and all 27 member states.

## Demarcating the Brussels Effect from the California and Beijing Effects

Bradford’s concept of the Brussels effect is based on the idea that disputes arising from different interpretations of key norms such as data privacy, free speech or fair market competition by the EU and its partners can be efficiently addressed by regulating private actors of the digital market. To secure access to the European market, private companies will design their terms of service in compliance with internal market standards and even lobby foreign governments to adopt legislation convergent with EU law in order to increase legal certainty; thus, the EU can also influence international legislative debates via private actors. Consequentially, the EU’s regulatory power in digital foreign policy is derived from its economic power, as evidenced by the fact that non-European digital technology companies — mainly headquartered in the US, but also in China — adjust their terms of services so that access to the European internal market is secured. The case study of the Brussels effect in action examining European policy-making by institutionalising and facilitating the TTC allows us to better understand key distinctions between the Brussels effect and the California and the Beijing effects, both of which are based on a similar assumption that their economic strength allows them to project power beyond their jurisdiction.

### Difference from the California Effect: Consensus Beyond Market Power

David Vogel has observed a “California effect” in the US, which saw states of the federation adopt certain environmental regulatory requirements to match the high standard of environmental protection necessary to be able to do business in California. California is the US state with the biggest GDP by far, surpassing Texas and New York by over 1.3 and 1.4 trillion USD, respectively (Bureau of Economic Analysis, 2022), what allowed externalising political objectives of the state via regulating access to its market (1995). Bradford coined the term “Brussels effect” in reference to this California effect, initially stating that she set out to explore “the dynamics of the California Effect in a global context” and identify conditions for successful norms externalisation as follows: “the jurisdiction must have a large domestic market, significant regulatory capacity, and the propensity to enforce strict rules over inelastic targets (e.g., consumer markets) as opposed to elastic targets (e.g., capital). In addition, unilateral regulatory globalization presumes that the benefits of adopting a uniform global standard exceed the benefits of adhering to multiple, including laxer, regulatory standards. This is the case in particular when the firms’ conduct or production is nondivisible, meaning that it is not legally or technically feasible, or economically viable, for the firm to maintain different standards in different markets” (2012).

Furthermore, Bradford provides ample empirical evidence that such an effect theorised as the California effect can be observed in a global context — namely the Brussels effect in the jurisdiction of the European Union (2012; 2020) — and points out that Brussels’ regulatory power is more pronounced than California’s, as Californian law still has to be consistent with US federal law. California is one state within the US and conducts its law-making in a bicameral system. The governorship, the state house of representatives and state senate are held by a Democratic trifecta since 2011, meaning that both chambers of congress have a majority for the Democratic Party and that the office of the governor is held by a member of the Democratic Party as well. Since 1999, the Republican Party could only succeed in winning the governorship from 2004 to 2010, but not achieve an assembly or senate majority (Ballotpedia, 2022). Furthermore, Republican governor Arnold Schwarzenegger made environmental sustainability a key topic of his agenda, matching ideological preferences of Californian voters but detouring from the environmental legislation strategy pursued by the GOP on the federal level and in other states (Weinberger, 2018). This means that the Democratic trifecta was able to pursue its legislative agenda rather easily, and far from having to reconcile positions as differing as across 27 EU member states.

The European Union is an organisation *sui generis*: One jurisdiction, but far from being one “bloc”. Ideological preferences, economic needs and political goals across member states vary, but previously agreed-on standards and minimum requirements of European legislative acts are expected to be broadly reflected in national law across the EU, and non-complying member states can be fined. Like California, the EU meets all of the criteria for successful norms externalisation as laid out by Bradford and is thus able to successfully leverage the Brussels effect. But we argue that there is also a further dimension equipping the Brussels effect with its considerable leverage: the political credibility derived from the fact that regulatory norms externalised by the Brussels effect have essentially already passed an intense vetting process of European debate and compromise in the multi-level governance system. EU policy-making involves not only all 27 member states — whose demands for legal requirements and broader ideological and strategic preferences often largely differ — but also a variety of European institutions and non-state actors. Thus, the EU as “one jurisdiction” can arguably generate international political capital expressed in the form of entering negotiations represented by the European Commission with a mandate explicitly given by the EU treaties.

### Difference from the Beijing Effect: Credibility Instead of Dependency

This internal dimension of the Brussels effect’s ability to generate political capital as a result of inter-level and inter-institutional debate which aids the European objective in international negotiations is also one of the factors that differentiate it from a purported “Beijing effect”. Matthew S. Erie and Thomas Streinz put forth their observation of a Beijing effect in 2021, theorising it as “a combination of push and pull factors that explains China’s growing influence in data governance beyond its borders.” They further observe that the Beijing effect contrasts with the Brussels effect insofar that the Chinese ability to provide technological know-how and equipment to developing economies — and thus, China’s technological and economic advantage — is the base condition for the Beijing effect to work, whereas the Brussels effect has demonstrated its efficiency towards national economies that are bigger than the European one and can boast the top providers of technological equipment and digital services worldwide. Additionally, the Beijing effect can both reinforce and undermine Chinese aspirations of externalising digital governance norms, as national governments can halt the development of technological and digital infrastructure by China (or with assistance from China) when they become too concerned of the effects of the Chinese National Intelligence Law, which mandates every citizen and every company to cooperate with national authorities by sharing information (that is, private data of costumers) or even manipulating technology it sells abroad (Stolton, 2019). Some EU Member States, the US and India are among jurisdictions that have banned some Chinese technology providers from participation in their otherwise free markets citing national security concerns (Erie & Streinz, 2021), thus undermining Chinese expansion efforts — both in economic terms and in norms externalisation. The Brussels effect, on the other hand, is not only based on hard market power, but also the ability to project its regulatory power via its legislative “strength of weakness” negotiated among 27 member states within a consensus-driven European decision-making process.

If access to such significant markets as the US or India is inhibited, Chinese reliance on the markets of developing countries increases. Such countries often (initially) welcome Chinese assistance in developing their technological and digital infrastructure, and therefore accept Chinese data governance norms to shape or even found their national data laws. This enables the Beijing effect to deliver an increase in overall connectivity and revenue, but in contrast to liberal projects, it does not advance citizens’ data sovereignty, freedom of expression or protection against hate speech, and thus can benefit society only economically. Furthermore, the Chinese legislative process is significantly less transparent and public than the European one, and legal certainty as the result of long inter-institutional and inter-level debate is not given as laws such as the Chinese National Intelligence Law can be adopted or amended quickly.

In summary, the California, Beijing and Brussels effects all are based on the core assumption that these jurisdictions can influence actors beyond their borders by setting regulatory standards. The effects differ insofar that:

- the Beijing effect is mainly efficient towards “weaker” and dependent actors, such as countries coerced into adapting Chinese standards to improve connectivity or receive loans, e.g. as granted by the Chinese Export–Import Bank;
- Unlike the California and Beijing effects, the Brussels effect is not only based on market power and technological capacities, but on credibility derived from the common standard-setting procedure of the European single market, a significant success story of European integration;
- The Brussels effect finally has an internal and external dimension that are mutually reinforcing. The Californian legislative process is steered by a Democratic trifecta, and the Chinese one is exclusively dominated by the authoritarian CPC. The EU comitology procedure entails debate and compromise necessary to find a consensus reconciling the needs and preferences of all member states. Internal agreement is a pre-condition for externalising norms, and knowing that externalisation is only possible when an agreement is given, the EU is piggybacking the political capital of compromise among member states in international negotiations. The TTC negotiations are thus a good case in point of how the external digital policy of the EU is deepening European integration as well.

## Conclusion and Policy Implications

In conclusion, the mutually reinforcing nature of the internal and external dimensions of the Brussels effect is based on European procedural sovereignty, which is a complex, multi-level process across nations and institutions, rooted in debate and founded on consensus and compromise. Europe’s “legal institutions” (Bradford, 2012) enable the Europeanisation of international standards and third parties’ regulatory policies, which benefits the EU and its citizens economically and socially. Thus, institutions and member states are incentivised to arrive at a consensus, as such a consensus is an important precondition for successful externalisation. This means that European policy-makers can leverage the Brussels effect to deepen integration and advance internal digital sovereignty, as they can argue that arriving at a compromise is necessary so that the European Commission can enter international negotiations with a mandate explicitly given by the EU treaties. Harmonised market regulations allow for more leverage and room for manoeuvre in international negotiations; thus, the Brussels effect as a political tool is not only based on market power, but also on the political credibility of the EU. The European decision-making process is complex and time-consuming, but it is also known to be transparent and legitimate. Hence, its results carry high legal certainty, and its tentative agreements reliably indicate strategic goals and political commitments. While Bradford herself reflects on internal motivations of the Brussels effect (2012), her considerations are limited to reflections on inter-European competitiveness and political preferences of member states which they seek to externalise in other member states; she does not consider the creation of political capital that can strengthen the EU position in international negotiations through internal norms harmonisation via the European decision-making process.

The four case studies conducted can confirm the analytical merit of the Brussels effect in the digital policy arena, assert its relevancy as a political tool and demonstrate that its concept has an additional and previously undiscussed level of being internally and externally mutually reinforcing. In the cases of cybersecurity, semiconductor value chain resilience and digital markets and digital services regulation, it is observable that European policy-makers set or propose standards concerning companies and markets largely beyond Europe, and that this (attempted) standard-setting provides the agenda for the transatlantic debate, as expected according to Bradford’s definitions of the Brussels effect (2012, 2020). “Open strategic autonomy” towards third states according to the European Commission is here within best safeguarded when embedded in an international network of democratic allies. The fourth case study on data protection regulation confirms the analytical value of the Brussels effect as well and demonstrates the mutually reinforcing nature of its internal and external dimensions ex negativo: the European failure to internally agree on guidelines for a viable replacement of the “privacy shield” negatively impacts American producers as well as European customers and clouds TTC negotiations.

The above findings bear policy implications as well: Currently, the European Union not only profits from the demonstrated enduring strength and relevance of the Brussels effect, but also from a US administration which seeks to intensify transatlantic cooperation. The EU has been forced to reconsider some of its policies given the lasting slow-down of production in China due to strict reactions to the COVID-19 pandemic and the Russian attack on Ukraine. Given the current lack of production capacities, know-how and human capital in the European Union, a paradigm shift from an ideal of “strategic autonomy” to a strategic narrative of “open strategic autonomy” has been set in motion, which understands that digital and technological sovereignty can only be achieved in cooperation with international partners. The first and foremost partner is the US, but other allies that are also bound to safeguard a rules-based international order and the protection of individual rights emerge on the map as well.

## Data Availability

Not applicable.

## References

[CR1] Alaily, R. & Klynge, C. (2021, December 10). Let’s stay focused and make the digital markets act effective. Microsoft EU Policy Blog. Retrieved 7 September 2022, from https://blogs.microsoft.com/eupolicy/2021/12/10/lets-stay-focused-and-make-the-digital-markets-act-effective/

[CR2] Breton, T. H. (2021, September 16). How a European Cyber Resilience Act will help protect Europe. EC Blog. Retrieved 7 September 2022, from https://ec.europa.eu/commission/commissioners/2019-2024/breton/blog/how-european-cyber-resilience-act-will-help-protect-europe_en

[CR3] Directive 2000/31/EC of the European Parliament and of the Council of 8 June 2000 on certain legal aspects of information society services, in particular electronic commerce, in the Internal Market (‘Directive on electronic commerce’) (2000, July 17). *Official Journal L 178.* Retrieved June 30, 2022, from http://data.europa.eu/eli/dir/2000/31/oj

[CR4] Directive (EU) 2016/1148 of the European Parliament and of the Council of 6 July 2016 concerning measures for a high common level of security of network and information systems across the Union (2016, July 19). *Official Journal of the European Union* L 194/1. Retrieved June 30, 2022, from http://data.europa.eu/eli/dir/2016/1148/oj

[CR5] European Economic and Social Committee. (2017, March 30). The digital single market - trends and opportunities for SMEs. Retrieved 11 November 2022, from https://www.eesc.europa.eu/en/our-work/opinions-information-reports/opinions/digital-single-market-trends-and-opportunities-smes-own-initiative-opinion

[CR6] European Parliamentary Research Service. (2020, July). Digital sovereignty for Europe. Retrieved 7 September 2022, from https://www.europarl.europa.eu/RegData/etudes/BRIE/2020/651992/EPRS_BRI(2020)651992_EN.pdf

[CR7] Federal Communications Commission. (2021, March 21). List of equipment and services covered by Section 2 of The Secure Networks Act. Retrieved 30 June 2022, from https://www.fcc.gov/supplychain/coveredlist

[CR8] H.R.7178 - 116th Congress (2019–2020): CHIPS for America Act, H.R.7178, 116th Cong. (2020, June 11). Retrieved 30 June 2022, from https://www.congress.gov/bill/116th-congress/house-bill/7178#:~:text=Introduced%20in%20House%20(06%2F11%2F2020)&text=This%20bill%20establishes%20investments%20and,manufacturing%20facility%20investment%20through%202026

[CR9] Judgment of the Court (Grand Chamber). (2020, July 16). Case C-311/18: *Data Protection Commissioner v Facebook Ireland Limited and Maximillian Schrems*. EU-Curia. Retrieved 30 June 2022, from https://curia.europa.eu/juris/document/document.jsf;jsessionid=3EE848D007055A4DAD963CA0DDC80B26?text=&docid=230683p&pageIndex=0&doclang=EN&mode=lst&dir=&occ=first&part=1&cid=14746278

[CR10] NIS Cooperation Group. (2019, October 09). EU coordinated risk assessment of the cybersecurity of 5G networks. Retrieved 30 June 2022, from https://digital-strategy.ec.europa.eu/de/node/1448

[CR11] NIS Cooperation Group. (2020, January 29). Cybersecurity of 5G networks - EU Toolbox of risk mitigating measures. Retrieved 30 June 2022, from https://digital-strategy.ec.europa.eu/en/library/cybersecurity-5g-networks-eu-toolbox-risk-mitigating-measures

[CR12] Proposal for a directive of the European Parliament and of the Council on measures for a high common level of cybersecurity across the Union, repealing Directive (EU) 2016/1148. *EUR-Lex* COM/2020/823 final (2020, December 16). Retrieved 30 June 2022, from https://eur-lex.europa.eu/legal-content/EN/TXT/?uri=COM%3A2020%3A823%3AFIN

[CR13] Proposal for a regulation of the European Parliament and of the Council on harmonised rules on fair access to and use of data (Data Act) (COM/2022/68 final) (2022, February 23). Retrieved 30 June 2022, from https://eur-lex.europa.eu/legal-content/EN/TXT/?uri=CELEX%3A52022PC0068&qid=1656858894570

[CR14] Proposal for a regulation of the European Parliament and of the Council on contestable and fair markets in the digital sector (Digital Markets Act) COM/2020/842 final (2020, December 15). Retrieved 30 June 2022, from https://eur-lex.europa.eu/legal-content/en/TXT/?qid=1608116887159&uri=COM%3A2020%3A842%3AFIN

[CR15] Proposal for a regulation of the European Parliament and of the Council on a Single Market For Digital Services (Digital Services Act) and amending Directive 2000/31/EC COM/2020/825 final (2020, December 15). Retrieved 30 June 2022, from https://eur-lex.europa.eu/legal-content/en/TXT/?qid=1608117147218&uri=COM%3A2020%3A825%3AFIN

[CR16] Regulation (EU) 2019/881 of the European Parliament and of the Council of 17 April 2019 on ENISA (the European Union Agency for Cybersecurity) and on information and communications technology cybersecurity certification and repealing Regulation (EU) No 526/2013 (Cybersecurity Act) (2019, June 07). *Official Journal of the European Union* L 151/15. Retrieved 30 June 2022, from http://data.europa.eu/eli/reg/2019/881/oj

[CR17] The White House (2022, October 07). Executive Order 14086/Executive Order on Enhancing Safeguards for United States Signals Intelligence Activities. Retrieved 8 November 2022, from https://www.whitehouse.gov/briefing-room/presidential-actions/2022/10/07/executive-order-on-enhancing-safeguards-for-united-states-signals-intelligence-activities/

[CR18] Alsop, T. H. (2022, July 27). Top semiconductor companies worldwide 2019–2021, by sales revenue. Statista. Retrieved 7 September 2022, from https://www.statista.com/statistics/283359/top-20-semiconductor-companies/#:~:text=Top%20semiconductor%20companies%20worldwide%202019%2D2021%2C%20by%20sales%20revenue&text=In%202021%2C%20Samsung%20retook%20market,sale%20of%20semiconductors%20during%202021

[CR19] Ballotpedia (2022). State Politics California. Retrieved 30 June 2022, from https://ballotpedia.org/California

[CR20] Baraniuk, C. (2021, August 27). Why is there a chip shortage? BBC News. Retrieved 30 June 2022, from https://www.bbc.com/news/business-58230388

[CR21] Bermingham, F. (2022, February 8). EU launches WTO case against China over Huawei, Xiaomi tech infringements. South China Morning Post. Retrieved 7 September 2022, from https://www.scmp.com/news/china/diplomacy/article/3167551/eu-launches-wto-case-against-china-over-huawei-xiaomi-tech

[CR22] Bendiek, A. (2021, April 02). The impact of the Digital Service Act (DSA) and Digital Markets Act (DMA) on European Integration Policy. SWP Working Paper. Retrieved 30 June 2022, from https://www.swp-berlin.org/publications/products/arbeitspapiere/WP0121_Bendiek_Digital_Service_Act_and_Digital_Markets_Act.pdf

[CR23] Bonß, W. (2021, February 01). *Was heißt „technologische Souveränität“? Aspekte einer Diskursentwicklung.* Forschungszentrum Risiko, Infrastruktur, Sicherheit und Konflikt der Universität der Bundeswehr München. Retrieved 30 June 2022, from https://132.230.133.75/images/pdf/aktuell/Wolfgang_Bon%C3%9F_-_Technologische_Souver%C3%A4nit%C3%A4t.pdf

[CR24] Bradford, A. (2012). The Brussels effect. Northwestern University Law Review, Vol. 107, No. 1, 2012. Columbia Law and Economics Working Paper No. 533, Available at SSRN: https://ssrn.com/abstract=2770634

[CR25] Bradford A (2020). The Brussels effect: How the European Union rules the world.

[CR26] Bhutada, G. (2021, December 14). The Top 10 semiconductor companies by market share. Visual Capitalist Vancouver. Retrieved 8 September 2022, from https://www.visualcapitalist.com/top-10-semiconductor-companies-by-market-share/

[CR27] Bureau of Economic Analysis (2022, June 30). Gross domestic product by state. US Department of Commerce. Retrieved 30 June 2022, from https://www.bea.gov/data/gdp/gdp-state

[CR28] Campbell, C. (2021, October 01). Inside the Taiwan firm that makes the world’s tech run. Time. Retrieved 8 September 2022, from https://time.com/6102879/semiconductor-chip-shortage-tsmc/

[CR29] Cerulus, L. (2020, February 28). How Huawei wields investment to bend European countries. Politico. Retrieved 8 September 2022, from https://www.politico.com/news/2020/02/28/huawei-investments-europe-118070

[CR30] Cerulus, L. & Barigazzi, J. (2021, September 29). France eyes control over chip agenda in EU-US tech alliance. Politico. Retrieved 8 September 2022, from https://www.politico.eu/article/france-eu-chips-strategy-control/

[CR31] Cervi, G. V. (2022, September 07). Why and how does the EU rule global digital policy: an empirical analysis of EU regulatory influence in data protection laws. *Digital Society* 1.18 (2022). 10.1007/s44206-022-00005-3

[CR32] Chee, F. (September 08, 2022). Draft EU rules target smart devices with cybersecurity risks. Reuters. Retrieved 8 September 2022, from https://www.reuters.com/technology/draft-eu-rules-target-smart-devices-with-cybersecurity-risks-2022-09-08/

[CR33] Ciani, A. & Nardo, M. (2022). The position of the EU in the semiconductor value chain: evidence on trade, foreign acquisitions, and ownership. JRC Working Papers in Economics and Finance. Retrieved 8 November 2022, from https://joint-research-centre.ec.europa.eu/system/files/2022-04/JRC129035.pdf

[CR34] Dachwitz, I. (2022, March 30). Das Privacy-Shield 2.0 ist zum Scheitern verurteilt. Netzpolitik. Retrieved 8 September 2022, from https://netzpolitik.org/2022/transatlantisches-daten-dilemma-das-privacy-shield-2-0-ist-zum-scheitern-verurteilt/

[CR35] Dluhosch B, Ziegler N (2011). The paradox of weakness in the politics of trade integration. Constitutional Political Economy.

[CR36] Echikson, B. (2022, July 12). Conclusion: bridging the digital divide. Center for European Policy Analysis. Retrieved 8 September 2022, from https://cepa.org/conclusion-bridging-the-digital-divide/

[CR37] Emmott, R. (2020, September 29). U.S. renews pressure on Europe to ditch Huawei in new networks. Reuters. Retrieved 8 November 2022, from https://www.reuters.com/article/us-usa-huawei-tech-europe-idUSKBN26K2MY

[CR38] Erie, M. S. & Streinz, T. (2021). The Beijing effect: China’s Digital Silk Road as Transnational Data Governance. *New York University Journal of International Law and Politics* 54.1. Retrieved 30 June 2022, from https://www.nyujilp.org/wp-content/uploads/2022/02/NYUJILP_Vol54.1_Erie_Streinz_1-91.pdf#page=1&zoom=auto,-22,67

[CR39] Erlanger, S. (2020, May 23). European Defense and “strategic autonomy” are also coronavirus victims. The New York Times. Retrieved 8 September 2022, from https://www.nytimes.com/2020/05/23/world/europe/defense-autonomy-europe-coronavirus.html

[CR40] European Parliament News. (2022, July 05). Digital Services: landmark rules adopted for a safer, open online environment. Retrieved 8 September 2022, from https://www.europarl.europa.eu/news/en/press-room/20220701IPR34364/digital-services-landmark-rules-adopted-for-a-safer-open-online-environment

[CR41] European Association of Automotive Suppliers. (2022, April 28). Adoption of EU Chips Act by early 2023 uncertain, as Parliament and Council start their work. Retrieved 8 September 2022, from https://clepa.eu/mediaroom/adoption-of-eu-chips-act-by-early-2023-uncertain-as-parliament-and-council-start-their-work/

[CR42] European Data Protection Board. (2022, March 22). Irish SA fines Meta Platforms (formerly Facebook) €17M for data breaches. Retrieved 30 June 2022, from https://edpb.europa.eu/news/national-news/2022/irish-sa-fines-meta-platforms-formerly-facebook-eu17m-data-breaches_en

[CR43] European Data Protection Board. (2021, July 22). Dutch DPA: TikTok fined for violating children’s privacy. Retrieved 30 June 2022, from https://edpb.europa.eu/news/national-news/2021/dutch-dpa-tiktok-fined-violating-childrens-privacy_en

[CR44] European Cloud Code of Conduct. (2022). Third Country Transfer Initiative. Retrieved 30 June 2022, from https://eucoc.cloud/en/about/third-country-transfer-initiative/

[CR45] European Commission. (2021a, March 09). Europe’s Digital Decade: digital targets for 2030. Retrieved 30 June 2022, from https://ec.europa.eu/info/strategy/priorities-2019-2024/europe-fit-digital-age/europes-digital-decade-digital-targets-2030_en

[CR46] European Commission. (2021b, October 18). EU-US Trade and Technology Council: Commission launches consultation platform for stakeholder's involvement to shape transatlantic cooperation. Retrieved 30 June 2022, from https://ec.europa.eu/commission/presscorner/detail/en/ip_21_5308

[CR47] European Commission. (2022). European Chips Act. Retrieved 30 June 2022, from https://ec.europa.eu/info/strategy/priorities-2019-2024/europe-fit-digital-age/european-chips-act_en

[CR48] Fitch, A., & Strumpf, D. (2019, December 01). Huawei manages to make smartphones without American chips. *The Wall Street Journal*. Retrieved 30 June 2022, from https://www.wsj.com/articles/huawei-manages-to-make-smartphones-without-american-chips-11575196201

[CR49] Greaves, P. & Nauwelaerts, W. (2022, March 25). EU and U.S. reach agreement in principle on a replacement for the EU-U.S. privacy shield. Alston & Bird. Retrieved 30 June 2022, from https://www.alstonprivacy.com/eu-and-u-s-reach-agreement-in-principle-on-a-replacement-for-the-eu-u-s-privacy-shield/

[CR50] Groothuis, B. (2022, November 10). Legislative Train Schedule: NIS 2. Retrieved 11 November 2022, from https://www.europarl.europa.eu/legislative-train/theme-a-europe-fit-for-the-digital-age/file-review-of-the-nis-directive

[CR51] Gkritsi, E. (2020, November 16). More European countries are turning their backs on Huawei. TechNode. Retrieved 7 September 2022, from https://technode.com/2020/11/16/insights-more-european-countries-are-turning-their-backs-on-huawei/

[CR52] Intel (2022, March 15). Intel announces initial investment of over €33 billion for R&D and manufacturing in EU. Intel Newsroom. Retrieved 30 June 2022, from https://www.intel.com/content/www/us/en/newsroom/news/eu-news-2022-release.html

[CR53] Komaitis, K., & Sherman, J. (2021, May, 11). US and EU tech strategy aren’t as aligned as you think. *Brookings Institution Tech Stream.* Retrieved 30 June 2022, from https://www.brookings.edu/techstream/us-and-eu-tech-strategy-arent-as-aligned-as-you-think/

[CR54] Kwan, C. (2022, February 13). Meta’s threat to leave Europe hints at waning big tech influence. ZDNet. Retrieved 30 June 2022, from https://www.zdnet.com/article/metas-threat-to-leave-europe-hints-at-big-tech-influence-waning/

[CR55] Le Corre, P. (2018) Chinese investments in European countries: experiences and lessons for the “Belt and Road” initiative. Carnegie Endowment. Retrieved 7 September 2022, from https://carnegieendowment.org/files/RethinkingtheSilkRoad.pdf

[CR56] Lee-Makiyama, H. & Baker, R. (2022, May 31). The US and Europe need to unite to build next generation 6G mobile networks. Otherwise, China will win. Center for European Policy Analysis. Retrieved 7 September 2022, from https://cepa.org/loosening-chinas-grip-on-telecommunications/

[CR57] Leonard, J., King, I., & Wu, D. (2022, June 13). China’s chipmaking power grows despite US effort to counter it. Bloomberg. Retrieved 7 September 2022, from https://www.bloomberg.com/news/articles/2022-06-13/china-s-growing-clout-in-global-chip-market-rings-alarm-bells-in-washington?leadSource=uverify%20wall

[CR58] Lewis, J.A., Conley, H.A., Wall, C., & Lostri, E. (2021, December). Choppy seas for a digital Atlantic. Center for Strategic and International Studies. Retrieved 30 June 2022, from https://csis-website-prod.s3.amazonaws.com/s3fs-public/publication/211214_Lewis_ChoppySeas_DigitalAtlantic.pdf?f2dwBFkdI7937s6HSCqpcnJB6CSjUGyB

[CR59] Köpsell, S., Ruzhanskiy, A., Hecker, A., Stachorra, D. & Franchi, N. (2022, Febraury 21). Open RAN risk analysis. German Federal Office for Information Security. Retrieved 7 September 2022, from https://www.bsi.bund.de/SharedDocs/Downloads/EN/BSI/Publications/Studies/5G/5GRAN-Risk-Analysis.pdf?__blob=publicationFile&v=7

[CR60] Larger, T. H., Scott, M., Kayali, L. & Vinocur, N. (2020, December 14). Inside the EU’s divisions on how to go after Big Tech. Politico. Retrieved 7 September 2022, from https://www.politico.eu/article/margrethe-vestager-thierry-breton-europe-big-tech-regulation-digital-services-markets-act/

[CR61] Lilkov, D. (2021, July 27). Securing Europe’s digital eastern front. *Center for European Policy Analysis*. Retrieved 30 June 2022, from https://cepa.org/securing-europes-digital-eastern-front/

[CR62] Lomas, N. (2021, September 15). Europe plans a Chips Act to boost semiconductor sovereignty. TechCrunch. Retrieved 30 June 2022, from https://techcrunch.com/2021/09/15/europe-plans-a-chips-act-to-boost-semiconductor-sovereignty/?guce_referrer=aHR0cHM6Ly9kdWNrZHVja2dvLmNvbS8&guce_referrer_sig=AQAAADwvoBLZMNoKpaLrPVHzZTsNIn83C61uHgrWGwkfUvJm07SgKgBCuIQK7eDPvVTb5L_Uu_KWNV6ukBecRDdcZSuI-Gb7hBv74cipamH0YLEkh-SPbZXOcwetbxkw4NkB1_-UWsBGZEo5Lq_BVLzwP6Xlzhzv9y6HoiOkYyy81fid&guccounter=2

[CR63] Mariniello, M., & Martins, C. (2021, December 14). Which platforms will be caught by the Digital Markets Act? The ‘gatekeeper’ dilemma. *Bruegel*. Retrieved 30 June 2022, from https://www.bruegel.org/2021/12/which-platforms-will-be-caught-by-the-digital-markets-act-the-gatekeeper-dilemma/

[CR64] Meyers, Z. (2021, May 04). Taming ‘Big Tech’: how the Digital Markets Act should identify gatekeepers. Centre for European Reform. Retrieved 30 June 2022, from https://www.cer.eu/insights/taming-big-tech-how-digital-markets-act-should-identify-gatekeepers

[CR65] Negreiro Achiaga, M. (2022, June 16). The NIS2 Directive. A high common level of cybersecurity in the EU. Think Tank of the European Parliament. Retrieved 30 June 2022, from https://www.europarl.europa.eu/thinktank/en/document/EPRS_BRI(2021)689333

[CR66] Niquet, V. (2018, March 07). Chinese Objectives in High Technology Acquisitions and Integration of Military and Civilian Capabilities: A Global Challenge. Note de la FRS. Retrieved June 30, 2022, from https://www.frstrategie.org/en/publications/notes/chinese-objectives-in-high-technology-acquisitions-integration-military-civilian-capabilities-global-challenge-2018

[CR67] Nocetti, J. (2019). Will europe remain a “digital colony”? *French Institute of International Relations.* Retrieved 16 November 2022, from https://www.ifri.org/sites/default/files/atoms/files/gomart_hecker_et_al_european_elections_2019_eng.pdf

[CR68] Noyan, O. (2021, November 05). Tech sector warns against politicisation of EU semiconductor industry. Euractiv. Retrieved 7 September 2022, from https://www.euractiv.com/section/digital/news/tech-sector-warns-against-politicisation-of-eu-semiconductor-industry/

[CR69] O’Brien, P. (2022, May 16). EU, US flaunt joint action to ‘hammer’ Russia on trade and tech. POLITICO. Retrieved 30 June 2022, from https://www.politico.eu/article/eu-us-joint-action-russia-trade-technology-council-summit/

[CR70] Pfeifle, S. (2018, October 25). US federal privacy law? Apple, Google, Facebook, Microsoft all hope so. International Association of Privacy Professionals. Retrieved 30 June 2022, from https://iapp.org/news/a/us-federal-privacy-law-apple-google-facebook-microsoft-all-hope-so/#

[CR71] Poitiers, N. (2021, September 22). Europe doesn’t need a ‘Mega-Fab.’ Bruegel. Retrieved 7 September 2022, from https://www.bruegel.org/comment/europe-doesnt-need-mega-fab

[CR72] Sacks, D. (2021, March 29). China’s Huawei is winning the 5G race. Here’s what the United States should do to respond. Council on Foreign Relations. Retrieved 7 September 2022, from https://www.cfr.org/blog/china-huawei-5g

[CR73] Salama, V. (2020, February 14). US won’t change intelligence sharing policy with UK despite Huawei decision. CNN Politics. Retrieved 7 September 2022, from https://edition.cnn.com/2020/02/14/politics/us-uk-intelligence-sharing/index.html

[CR74] Scharpf, F. W. (1999). *Regieren in Europa. Effektiv und demokratisch?* (Frankfurt/Main: Campus). https://www.mpifg.de/173989/mpifg_sbd_fs1999.pdf

[CR75] Schoolov, K. (2022, March 23). Inside TSMC, the Taiwanese chipmaking giant that’s building a new plant in Phoenix. CNBC News. Retrieved 30 June 2022, from https://www.cnbc.com/2021/10/16/tsmc-taiwanese-chipmaker-ramping-production-to-end-chip-shortage.html

[CR76] Scott, M. & Manancourt, V. (2022, March 24). US eyes breakthrough on data dispute with EU as Biden visits Brussels. Politico. Retrieved 7 September 2022, from https://www.politico.eu/article/us-eyes-breakthrough-on-data-dispute-with-eu-biden-visit-privacy-shield-ukraine/

[CR77] Shead, S. (2022, February 07). Meta says it may shut down Facebook and Instagram in Europe over data-sharing dispute. CNBC. Retrieved 30 June 2022, from https://www.cnbc.com/2022/02/07/meta-threatens-to-shut-down-facebook-and-instagram-in-europe.html

[CR78] Stolton, S. (2022, January 31). US pushes to change EU’s digital gatekeeper rules. POLITICO. Retrieved 30 June 2022, from https://www.politico.eu/article/us-government-in-bid-to-change-eu-digital-markets-act/

[CR79] Stolton, S. (2019, April 16). Huawei admits Chinese law obliges companies to work with government, under conditions. EURACTIV. Retrieved 30 June 2022, from https://www.euractiv.com/section/cybersecurity/news/huawei-admit-chinese-law-obliges-companies-to-work-with-government/

[CR80] Terharen, F. (2022, February 10). Landmark decision in Austria: use of Google Analytics found to breach GDPR. Schönherr Associates. Retrieved 30 June 2022, from https://www.schoenherr.eu/content/landmark-decision-in-austria-use-of-google-analytics-found-to-breach-gdpr/

[CR81] Tekdeeps (2020, July 11). *Tekdeeps* Spain questions for the first time the security of Huawei. Retrieved 7 September 2022, from https://tekdeeps.com/spain-questions-for-the-first-time-the-security-of-huawei/

[CR82] Tiku, N. (2018, March 19). Europe’s new privacy law will change the web, and more. *Wired.* Retrieved 30 June 2022, from https://www.wired.com/story/europes-new-privacy-law-will-change-the-web-and-more/

[CR83] Torreblanca, J. I., & R. Jorge Ricart (January 2022). The US-EU Trade and Technology Council (TTC): state of play, issues and challenges for the transatlantic relationship. Esade EcPol Center for Economic Policy. Retrieved 7 September 2022, from https://www.esade.edu/ecpol/wp-content/uploads/2022/12/AAFF_EcPol-OIGI_PaperSeries_ENG_def_jan22.pdf?_gl=1*itg2cz*_up*MQ..*_ga*NDQzMDk1Mzg1LjE2NjMwNTc1Nzg.*_ga_S41Q3C9XT0*MTY2MzA1NzU3OC4xLjAuMTY2MzA1NzU3OC4wLjAuMA

[CR84] UN Conference on Trade and Development. (2021). Technology and innovation report 2021. Retrieved 30 June 2022, from https://unctad.org/system/files/official-document/tir2020_en.pdf

[CR85] Vogel D (1995). Trading up: Consumer and environmental regulation in a global economy.

[CR86] Weinberger, J. (2018, January 8). How Arnold Schwarzenegger is saving the environment. CNBC News. Retrieved 7 September 2022, from https://www.cnbc.com/2018/01/08/how-arnold-schwarzenegger-is-saving-the-environment.html

